# Indigenous Knowledge of the Traditional Use of Aromatic and Medicinal Plants in Rif Mountains Ketama District

**DOI:** 10.1155/2023/3977622

**Published:** 2023-07-29

**Authors:** Yahya El-Mernissi, Aziz Zouhri, Amina Labhar, Naoual El Menyiy, M'hamed Ahari, Soufian El Barkany, Amin Salhi, Abdelhakim Bouyahya, Lhoussain Hajji, Hassan Amhamdi

**Affiliations:** ^1^Research Unit in Applied Chemistry, Faculty of Sciences and Techniques, Abdelmalek Essaadi University, Al-Hoceima 32003, Morocco; ^2^Laboratory of Pharmacology, National Agency of Medicinal and Aromatic Plants, Taounate 34025, Morocco; ^3^Bioactives and Environmental Health Laboratory, Faculty of Sciences, Moulay Ismail University, B.P 11201, Meknes, Morocco; ^4^Laboratory of Molecular Chemistry, Materials and Environment (LMCME), Department of Chemistry Faculty Multidisciplinary Nador, Mohamed 1st University, P.B 300, Nador 62700, Morocco; ^5^Laboratory of Human Pathologies Biology, Department of Biology, Faculty of Sciences, Mohammed V University in Rabat, Rabat 10106, Morocco

## Abstract

**Background:**

Medicinal plants have long played an integral role in traditional healing systems and are crucial for meeting primary healthcare needs. This study aimed to investigate the use of medicinal plants in phytotherapy in the Ketama region of Northern Morocco.

**Methods:**

Ethnobotanical data and ancestral knowledge regarding plants were collected through a field survey conducted from August 2019 to July 2021. The data were gathered using a standardized questionnaire, as well as through semistructured interviews and focus groups. Various ethnobotanical indices were applied to analyse the information collected.

**Results:**

A comprehensive inventory identified a total of 81 plant species, belonging to 40 families and 65 genera. These species are used primarily to treat a variety of diseases. Notably, digestive disorders ranked first among the diseases treated, with an ICF value of 0.618. *Rosmarinus officinalis* L., *Thymus serpyllum* L., and *Origanum compactum* Benth exhibited the highest UV values among medicinal plants. Leaves were the most used part of the plant part (50.28%), and the decoction method was the most recommended preparation, with oral administration being the preferred mode of application of the remedy.

**Conclusion:**

The Ketama region boasts a rich abundance of medicinal and aromatic plants, as evident from the quantitative analysis highlighting the significant usage of *Rosmarinus officinalis* L., *Thymus serpyllum* L., and *Origanum compactum* Benth. by the local population. However, further research in the form of pharmacological studies is necessary to validate their therapeutic effects.

## 1. Introduction

The use of medicinal plants has been an integral part of traditional healthcare systems since antiquity. Approximately 391,000 plant species are distributed throughout the world, each with its repertoire of medicinal properties, of which 88 percent (31,000) have been identified as being either used or potentially therapeutic plant species are distributed throughout the world, each with its repertoire of medicinal properties, of which 88 percent (31000) have been identified as being either used or potentially therapeutic [1].

Various modes of application are adapted by indigenous populations to the use of medicinal plants. Although many new medicines are derived from plants, approximately 60–80% of the population in developing countries still rely on herbal medicines [2, 3].

Several factors contribute to the use of herbal medicines and herbal remedies in developing countries. These include cultural acceptance, ease of availability, and cost efficiency compared to synthetic medications.

Due to its geographical location, Morocco has a very rich ecological and plant diversity, forming a real botanical [4] reserve with nearly 3913 species belonging to 981 genera and 155 families [5].

Multiple ethnobotanical surveys have been carried out in the Rif region to document and record traditional medicinal plant use practices [6–12]. However, the Rif region is still not explored ethnobotanically enough, specifically the Ketama area, due to geographical constraints such as high terrain and slopes, as well as cultural restrictions that limit researchers' access to documenting medicinal plant practices. The local community in the area is bound by ancient traditions and customs.

The aim of this study was to identify the species of medicinal plants and the traditional use of medicinal plants used in Ketama, as well as to gather information on traditional remedies for various illnesses. The study also aimed to document the plant parts used, preparation methods, and treatments used in these remedies.

## 2. Materials and Methods

### 2.1. Description of the Study Area

**Figure 1:**
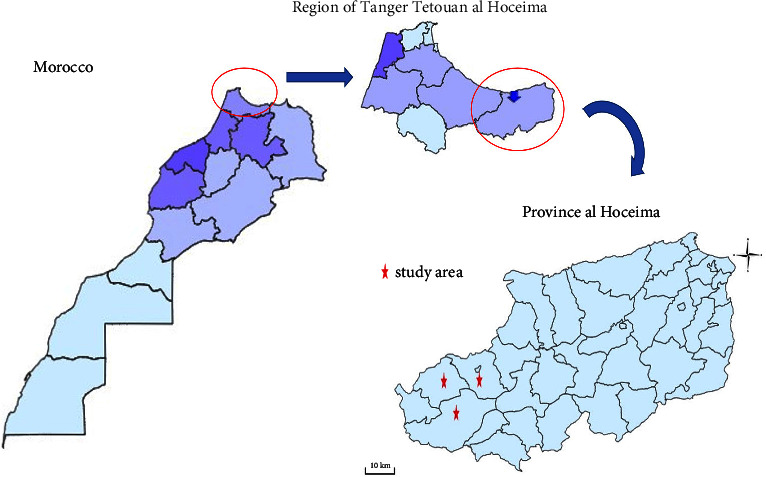
Geographical location of the study area.

This survey was conducted in the Ketama region (northern Morocco), approximately 431 km from Rabat, the administrative capital of Morocco. Ketama is an area that is part of the province of Al-Hoceima; it is located on the Mediterranean coast and belongs to the Tangier-Tetouan-Al Hoceima region following the 2015 territorial division. The study area is located at 34° 52′ 35″ N. 4° 37′ 10″ W, and it has a total population of 45683 people according to the 2015 census report [13]. The Ketama region consists of four localities: Ketama, Tamsaout, Abdelghaya Souahel, and Issaguen. Bounded to the south by the province of Taounate, and to the west by the province of Chefchaouen. The study area is characterized by an abundance of flowers and a mountainous geography with an average altitude of 1115 meters (Figure 1). It has a warm Mediterranean climate with dry summers. With an average temperature of 18.6°C and an average annual rainfall of 642.1 mm, agriculture is the principal economic activity in this area, and its products are the main source of living for the population. These products are mainly based on subsistence agriculture which includes livestock, arboriculture, and cereals.

### 2.2. Data Collection

This ethnobotanical survey was conducted over a period from August 2019 to July 2021 using simple random sampling. 10 field trips were conducted to collect ethnobotanical information. A total of 352 informants are included in the study, and all are native to the study area and were interviewed using an open-ended semistructured questionnaire that included on the one-hand general data about the informant such as age, gender, family status, place of residence, education level and, on the other hand, his knowledge of herbal medicine, the local name of the plant, the parts used in treatments, the diseases treated, the methods of preparation, the route of administration, the quantity used per day, the duration of use, and whether this plant has side effects or toxicity. The interviews were conducted in Darija (Moroccan dialect). According to our state regulations, the collection of ethnobotanical data does not require ethics approval. However, the documentation of medicinal uses obtained from the local population in the study area was acknowledged with thanks. This study followed and incorporated the recommendations made by Heinrich et al. [14] for field methods to conduct ethnopharmacological research.

### 2.3. Plant Identification and Conservation

The plant species were carefully pressed and put on herbarium sheets following the techniques of Martin [15]. Taxonomic identification was performed using the flora of Morocco [16] and the catalogue of vascular plants of northern Morocco [17]. The exact botanical name was obtained from The World Flora Online (https://www.worldfloraonline.org) and The Plant List (https://www.theplantlist.org). The samples of plants were authenticated in the herbarium of medicinal and aromatic plants of the National Agency of Medicinal and Aromatic Plants in Taounate.

### 2.4. Quantitative Data Analysis

#### 2.4.1. Informant Consensus Factor (ICF)

One of the indices utilized to assess the consistency of the information provided by informants. The formula presented by [18] was utilized to determine this index.

(1) $$\begin{array}{c} ICF=\frac{N_{ur}-N_{t}}{N_{ur}-1}\text{,} \end{array}$$

where *N*_*ur*_ is the total number of use reports cited for each disease category, and *N*_*t*_ is the total number of taxa used in that disease category. The ICF value ranges from 0 (if informants do not communicate use information) to 1 (if informants are found to exchange their knowledge).

#### 2.4.2. Frequency of Citation (FC)

The frequency of citation allows us to assess the credibility of the information received and the level of knowledge of the plants of the surveyed population [19]. The frequency of citation (FC) of a species corresponds to the number of informants who cited the species.

#### 2.4.3. Relative Frequency of Citation (RFC)

The relative frequency of citation (RFC) has been calculated to assess the level of agreement among informants on the declared species. This index's role is to demonstrate the local importance of each species [19], which is expressed as follows:

(2) $$\begin{array}{c} RFC=\frac{FC}{N}\text{,} \end{array}$$

where FC is the frequency of citation, and *N* is the total number of informants in the survey.

#### 2.4.4. Fidelity Level (FL)

Friedman et al. proposed the fidelity level (FL) to analyse plant use among Bedouins [20]. As defined by FL, it is the ratio of informants who independently suggested that a species be used for the same major purpose to all informants who mentioned the plant for any reason.

To calculate the FL index, the following formula is used:

(3) $$\begin{array}{c} FL=\frac{Ip}{Iu}\times 100\text{,} \end{array}$$

*where Ip* is the number of informants who independently indicated the use of a species for the same major ailment, and *I*_*U*_ is the total number of informants who mentioned a plant for any major ailment.

#### 2.4.5. Use Value (UV)

Is an index proposed by Philips and Gentry in 1993 to quantify the importance of species [21], UV is calculated according to the formula reported by Albuquerque [22].

(4) $$\begin{array}{c} UV=\frac{\sum U_{i}}{N}\text{,} \end{array}$$

where *U*_*i*_ is the number of uses mentioned by each informant, and *N* is the total number of informants.

#### 2.4.6. Family Use Value (FUV)

The family importance value is used to estimate the importance of medicinal plant families, within the informants [23]. This index is calculated by the formula given by [24].

(5) $$\begin{array}{c} FUV=\frac{{UV}_{S}}{Ns}\text{,} \end{array}$$

where UV_S_ is the number of informants mentioning the family, and *Ns* is the total number of species in each family.

#### 2.4.7. Jaccard Index (JI)

Ethnobotanists estimate the Jaccard index to compare data collected to previously published data obtained from bordering sites.

(6) $$\begin{array}{c} JI=\frac{c\times 100}{\left(a+b\right)-c}\text{,} \end{array}$$

where a is the number of species found in area *A*, *b* is the number of species found in area *B*, and *c* is the number of species found in area A and area B [25].

### 2.5. Data Analysis

The medicinal plants reported in this survey were classified alphabetically in table form according to their family, and the following data were listed: scientific name, local name, parts used, preparation, and diseases treated. The quantitative data frequency of citation (FC), relative frequency of citation (RFC), family use value (FUV), use value (UV), fidelity level (FL), and Jaccard index (JI) were analysed and summarized as proportions or percentages using descriptive statistics in Microsoft Excel 356.

## 3. Results and Discussion

### 3.1. Demographic Descriptions of the Informants

The study lasted approximately 2 years, from 2019 to 2021. During this period, 10 field trips were conducted to collect ethnobotanical information on the use of plants in different areas of the Ketama district. A total of 352 informants, all native to the study area, were included in the study. More than half of the informants were women (52.7%) and men (47.73%), which can be partly explained by the fact that women use plants more than men; however, also, we can assume that women play a more important role in ethnomedical practices. The age group of 40–50 years is the most present in the study population, followed by the 50–60 years age group with 23.58% against 19.60% their age between 30 and 40 years, while the age groups less than 30 years and more than 60 years are about 9.38% and 20.17%, respectively (Table 1). The maximum knowledge about the use of medicinal plants was obtained from informants aged between 40 and 60 years. However, informants under 40 years of age provided the minimum knowledge. In most ethnobotanical studies, increasing urbanization and a lack of initiatives to document indigenous knowledge have resulted in the deterioration of indigenous knowledge, indicating the knowledge gap between the older and younger generation in terms of knowledge sharing. The same result was reported by Chaachouay et al. [8]. A large number of the informants are illiterate (63.64%), while the informants who attended elementary school (24.43%) and secondary school (11.36%) are also among those who obtained university degrees (0.57%). A large gap in educated informants was observed in the study area. This is due to the lack of educational facilities, especially secondary and university schools in the study area. Most of the informants are married (86.65%) and unmarried (13.35%). All informants speak Darija, a dialectal language in Morocco, and Rifiya, an Amazigh language specific to the Rif people.

### 3.2. Medicinal Plant Diversity

During the ethnobotanical survey, 81 plant species and 65 plant genera from 40 families were documented. As used by the residents of the Ketama district in Al-Hoceima province. The results obtained, containing a detailed account of the botanical name of the plant, the locale name, the medicinal uses, the part(s) used, preparation, and FC, RFC, UV, FUV indices, are presented in Table 2.

**Figure 2:**
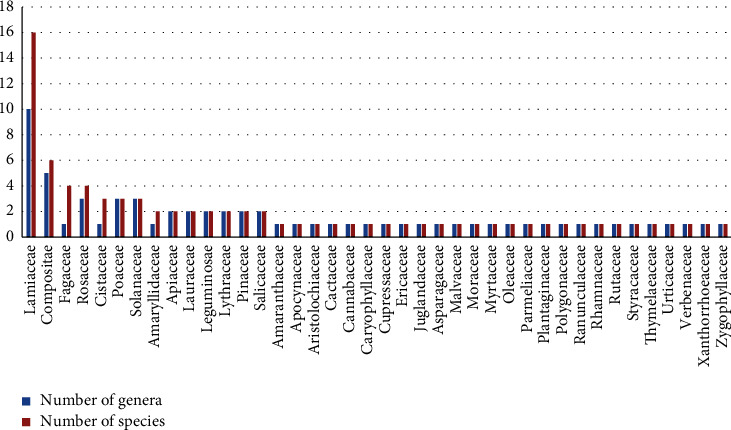
Plant families of medicinal plants used in Ketama.

The Lamiaceae family was the most used (16 species, 10 genera), followed by the Compositae (6 species, 5 genera, FUV = 0.026), Rosaceae (4 species, 3 genera, FUV = 0.011), and Fagaceae (4 species, 1 genera, FUV = 0.006). The other families that are frequently employed were the Cistaceae (3 species, 1 genera), Poaceae, Solanaceae (3 species, 3 genera), Apiaceae, Lauraceae, Leguminosae, Lythraceae, Pinaceae, Salicaceae (2 species, 2 genera each family), Amaryllidaceae (2 species, 1 genus), and Amaryllidaceae (2 species, 1 genus). On the other hand, Amaranthaceae, Apocynaceae, Aristolochiaceae, Cactaceae, Cannabaceae, Caryophyllaceae, Cupressaceae, Ericaceae, Juglandaceae, Liliaceae, Malvaceae, Moraceae, Myrtaceae, Oleaceae, Parmeliaceae, Plantaginaceae, Polygonaceae, Ranunculaceae, Rhamnaceae, Rutaceae, Styracaceae, Thymelaeaceae, Urticaceae, Verbenaceae, Xanthorrhoeaceae, and Zygophyllaceae were represented by one species per family (Figure 2).

In the present study, the largest family by number of taxa is Lamiaceae (16 species). Similarly, other studies conducted in the province of Al-Hoceima, and other provinces of Morocco [6, 8, 26] reported the predominance of the Lamiaceae, and this is in accordance with our findings. Herbaceous life forms may contribute to the dominance of this family and its wide distribution [5]. Also, the dominance of these families can be attributed to their abundance in the flora of the study area and the flora of Morocco.

### 3.3. Part Used, Method of Preparation, and Administration of Medicinal Plants Used to Cure Diseases

**Figure 3:**
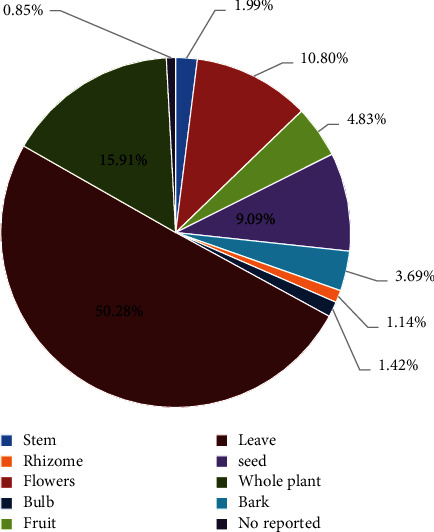
Frequency of parts used of medicinal plants in Ketama.

According to the results obtained (Figure 3), most parts of plants used in the preparation of herbal remedies are leaves (50.28%) followed by the whole plant (15.91%), flowers (10.80%), and seeds (9.09%), while the usage percentage of other plant parts is less than five percent. In other similar studies [8, 27–29] conducted in different regions of Morocco and the world, it was found that the leaf is the most used part of the plant. The researchers believe that the leaves are commonly used in herbal medicine because they are easy to collect and because they are photosynthetic and therefore contain more secondary metabolites than other parts of the plant [30].

**Figure 4:**
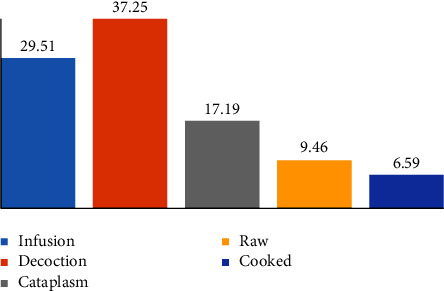
Modes of preparation of medicinal plants in Ketama.

The plants mentioned by respondents were mainly used in the form of decoction (37.25% of the responses), infusion (29.51%), and cataplasm (17.19%). Other uses (raw and cooked) are less common (Figure 4). In comparison with other studies conducted in different parts of the world, our results are similar [31–33]. As a method of extracting herbal remedies using water and other liquids, namely honey and olive oil, the decoction was found to be the most common form of herbal preparation in ethnobotanical studies [26].

**Figure 5:**
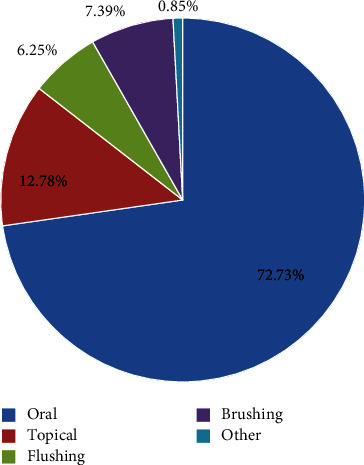
Different modes of administration of plant-based therapeutic preparation.

More than half of herbal remedies are taken orally (72.73%), which is the main route of administration. 256 preparations were taken by oral route, and 45 preparations (12.78%) were used to treat a variety of disorders. The percentage of the other route for the administration group does not exceed 14.49% (Figure 5).

In agreement, the use of the oral route is widespread among ethnic groups in different regions of Morocco, as found in our study. Furthermore, topical application is also an important route of administration of herbal remedies used in the treatment of various external diseases, including wounds, rheumatism, skin disorders, and muscle pain, as has been reported in many previous studies [26, 34, 35].

### 3.4. Quantitative Analysis

#### 3.4.1. Informant Consensus Factor ICF

ICF is an index used to evaluate the agreement of informants on the way to treat diseases, and the diseases treated by plants in this survey are divided into 8 categories. The findings of the present survey (Table 3) show that the ICF value ranges from 0.618 to 0.133. Digestive diseases and respiratory diseases have the highest ICF (0.618 and 0.451), respectively, followed by musculoskeletal diseases (0.486), dermatological diseases (0.423), endocrine, metabolic, and nutritional diseases (0.333), urological diseases (0.313). On the other hand, the disease categories with the lowest ICF were cardiovascular disease (0.222) and nervous system disease (0.133).

In the study area, we frequently observed that informants who used plants to treat digestive and respiratory diseases had the highest ICF compared to other disease categories; the same result is reported in other previous studies conducted in the north and south of Morocco [28–30, 36, 37]. This can be explained by the fact that the plants used to treat ailments are well-known among informants in the study area to treat ailments of this nature.

#### 3.4.2. Relative Frequency of Citation (RFC) and Frequency of Citation (FC)

The value of the RFC varied between 0.004 and 0.139, and the species with the highest RFC value was *Thymus serpyllum* L. followed by *Mentha pulegium* L. and *Origanum compactum* Benth. The species with the lowest RFC are the species that have been mentioned only once, which are *Crataegus monogyna* Jacq. *Anacyclus pyrethrum* (L.) Lag (Table 2).

It is evident that the medicinal plant species are most frequently used for the management of specific diseases by the local population. The FC and RFC allow us to determine the most cited and therefore the best-known medicinal plants in the study area, according to the results obtained *Thymus serpyllum* L. is the best-known species for the treatment of various diseases. Several studies on *Rosmarinus officinalis* L. have been conducted using biological and pharmacological approaches, revealing antispasmodic, antibacterial, and antioxidant properties [38].

#### 3.4.3. Fidelity Level of Medicinal Plants

The value of FL varies from 2.8 to 100 percent in this study, and species with FL equal to 100% are used to treat only one category of disease.

The fidelity level calculation allows us to know the main therapeutic use of each species, and a higher fidelity level indicates that the plants are used only for the treatment of diseases. In this study, digestive diseases are treated by 44 species but only 12 species among them are only against digestive diseases, e.g., *Arbutus unedo* L. while *Marrubium vulgare* L., *Mentha  ×   rotundifolia* (L.)Huds., and *Thymus vulgaris* L. have a 100% FL since they are used as a remedy only against respiratory diseases. In the Middle Atlas of Morocco, *Marrubium vulgare* L. is used in the therapy of respiratory diseases [27] as shown in Table 4.

#### 3.4.4. Use Value of Species and Families

In the present study, the UV index of the recorded botanical species ranged from 0.004 to 0.044 (Table 2). The highest UV index was calculated for Rosmarinus officinalis L., while Thymus serpyllum L. and Origanum compactum Benth. showed the same value (UV = 0.044). They were followed by Dittrichia viscosa (L.) Greuter with a UV index of 0.036, and Cannabis sativa L. with a UV index of 0.032. However, low-citation species and their respective values were *Dysphania ambrosioides* (L.) Mosyakin & Clemants, *Allium cepa* L. (UV = 0.004) (Table 3).

The calculation of use value gives an idea about the use of the species. Some species have a higher UV than others because of their frequent use in the treatment of various diseases. Due to their reputation as natural remedies with fewer side effects, they are well-known among the population [39]. In this study, *Rosmarinus officinalis* L., *Thymus serpyllum* L. *Origanum compactum* Benth., the most used species in the Ketama area are generally used in all Morocco [31, 32, 40, 41], as well as in North Africa [42].

This explanation clarifies the use of *Rosmarinus officinalis* L. in traditional medicine. This plant is abundant in secondary metabolites, such as phenolic acids, flavonoids, and alkaloids, which could potentially exhibit efficacy in the treatment of various diseases. Notably, two prominent diterpenes, carnosic acid and carnosol, have been identified in *Rosmarinus officinalis* L. [43]. These compounds possess antioxidant properties through their electron-donating capacity, thus protecting lipid membranes against oxidative damage [44].

*Thymus serpyllum* L., commonly known as thyme, contains essential oils with a notable composition of 36.5% thymol. Thymol is a pharmacologically active compound that imparts antioxidant, antimicrobial, and anti-inflammatory effects [45].

In the case of *Origanum compactum* L., also called compact oregano or Moroccan oregano, the principal compound is carvacrol. Carvacrol, a phenolic compound, is responsible for the characteristic aroma and flavour of oregano. Extensive research has been conducted on carvacrol, revealing its antimicrobial, antioxidant, anti-inflammatory, and anticancer activities. As one of the primary bioactive constituents in *Origanum compactum* L., carvacrol significantly contributes to its traditional medicinal applications [46].

#### 3.4.5. Jaccard Index JI

The Jaccard similarity index (Table 5) was calculated for 10 published studies, and the criterion used for the choice of the study is that the location of the region is close to the location of our study. The results revealed that the Taounate region is the most similar to the Ketama region with JI = 36.78 followed by the eastern region (JI = 26.77) and Ksar Lakbir (JI = 22.64). However, the study with the lowest similarity is the one conducted in Talassemtane National Park (JI = 1.09), and the other studies' JI is between 18.91 and 4.04.

The results revealed that the province of Taounate was the most comparable to Ketama. This can be attributed to several factors. First, the geographical aspect plays a role, as Taounate province represents the southern limit of Ketama. In addition, the socio-economic and socio-cultural contexts are influential factors. The Ketama area serves as the workplace for numerous farmers from Taounate, and there is also a significant population in both Taounate and Ketama belonging to the Jebala ethnic group. However, the lowest IJ is that of the Talassemtane area, which is isolated and difficult to access. We know that ethnobotanical knowledge may be influenced by isolation in the mountains [47], which decreases the sharing and transmission of ethnobotanical knowledge and practices.

## 4. Conclusion

Traditional medicine plays a vital role in the healthcare system of many developing countries, which is highly dependent on medicinal flora. This study aimed to document the potential medicinal plants in the Ketama area of Al-Hoceima province, representing the first ethnobotanical survey conducted in this region. The survey revealed a remarkable richness, with 81 different medicinal plants from 40 families and 65 genera identified. Among them, *Rosmarinus officinalis* L., *Thymus serpyllum* L., and *Origanum compactum* Benth. are emerged as the most widely recognized medicinal plants by the local population, based on the UV index. However, further research, including pharmacological and phytochemical analysis such as the isolation of bioactive compounds, is necessary to confirm their traditional use. This study also highlights the significant traditional knowledge passed down through generations, providing valuable information about the Ketama area. Moving forward, prioritizing research efforts to investigate the therapeutic potential and safety of these medicinal plants is essential.

## Figures and Tables

**Table 1:** Demographic data of informants.

Demographic features	Category	Number	Percentage
Sex	Female	184	52.27
	Male	168	47.73

Age	<30 years	33	9.38
	30–40 years	69	19.60
	40–50 years	96	27.27
	50–60 years	83	23.58
	>60 years	71	20.17

Educational level	Illiterate	224	63.64
	Primary	86	24.43
	Secondary	40	11.36
	University	2	0.57

Marital status	Not married	47	13.35
	Married	305	86.65

**Table 2:** List of plants used by the population of Ketama.

Scientific names of species and families	Parts used	Preparation	Medicinal uses	FC	RFC	∑*U*_*i*_	UV	FUV
Amaranthaceae								0.004
*Dysphania ambrosioides* (L.) Mosyakin & Clemants	Leaf	Raw	Sedative	1	0.004	1	0.004	
Amaryllidaceae								0.006
*Allium cepa* L.	Bulb	Raw	Hypotensive	1	0.004	1	0.004	
*Allium sativum* L.	Bulb	Raw	Hair care, headache	2	0.008	2	0.008	
Apiaceae								0.010
*Petroselinum crispum* (Mill.) Fuss	Leaf	Infusion, decoction	Stomachic, kidney pains, intestinal pains	3	0.012	3	0.012	
*Foeniculum vulgare* Mill	Seed	Infusion, cooked	Rheumatic pains, intestinal pains	2	0.008	2	0.008	
Apocynaceae								0.024
*Nerium oleander* L.	Stem, powder, rhizome, leaf	Decoction, cataplasm	Cutaneous infections, relaxant, skin wounds, antiseptic, rheumatic pains, intestinal pains	6	0.024	6	0.024	
Aristolochiaceae								0.012
*Aristolochia longa* L.	Leaf, whole plant	Infusion, cataplasm, raw	Cutaneous infections, diabetes, hypoglycemic	5	0.020	3	0.012	
Asparagaceae								0.004
Drimia maritima (L.) Stearn.	Bulb	Raw	Ocular infections	1	0.004	1	0.004	
Cactaceae								0.012
*Opuntia ficus-indica* (L.) Mill.	Flowers, fruit, bark	Decoction, cataplasm, raw	Skin ulcers, urinary infections, intestinal pains	3	0.012	3	0.012	
Cannabaceae								0.032
*Cannabis sativa* L.	Flowers, seed, leaf, whole plant	Catapalsm, raw, cooked	Skin protection, hair tonic, respiratory infections, sedative, cancer, hypoglycemic, hypotensive, hair care	13	0.052	8	0.032	
Caryophyllaceae								0.004
*Herniaria hirsuta* L.	Leaf, whole plant	Infusion	Against kidney stones	3	0.012	1	0.004	
Cistaceae								0.008
*Cistus albidus* L.	Seed, leaf	Infusion, decoction, cataplasm	Intestinal pains, respiratory infections, cutaneous infections, hypotensive, hypoglycemic	12	0.048	5	0.020	
*Cistus ladanifer* L.	Seed, leaf	Infusion, decoction	Intestinal pains	3	0.012	1	0.004	
*Cistus laurifolius* L.	Seed, leaf	Cooked	Hypotensive	3	0.012	1	0.004	
*Cistus salviifolius* L.	Seed, leaf	Infusion, decoction	Gastric pains	2	0.008	1	0.004	
Compositae								0.013
*Anacyclus pyrethrum* (L.) Lag.	Whole plant	Raw	Intestinal pains	1	0.004	1	0.004	
*Artemisia arborescens* (Vaill.) L.	Leaf	Infusion	Gastric pains, sedative	2	0.008	2	0.008	
*Artemisia herba-alba* Asso	Stem, flowers, leaf	Infusion, decoction, cataplasm, cooked	Skin wounds, skin protection, rheumatic pains, intestinal pains, hypotensive	7	0.028	5	0.020	
*Cyanus triumfettii* (All.)	Stem	Decoction	Rheumatic pains	1	0.004	1	0.004	
*Dittrichia viscosa* (L.) Greuter	Flowers, leaf, whole plant	Decoction, cataplasm, cooked	Gastric pains, skin wounds, respiratory infections, rheumatic pains, skin ulcers, asthma, skin protection, cutaneous infections, hypotensive	14	0.056	9	0.036	
*Scolymus hispanicus* L.	Stem	Cooked	Intestinal pains	1	0.004	1	0.004	
Cupressaceae								0.016
*Tetraclinis articulata* (Vahl) Mast.	Leaf	Infusion, decoction	Skin wounds, antipruritic, rheumatic pains, cold	4	0.016	4	0.016	
Ericaceae								0.008
*Arbutus unedo* L.	Flowers, fruit	Decoction, cataplasm	Gastric pains, intestinal pains	2	0.008	2	0.008	
Fagaceae								0.006
*Quercus faginea* Lam.	Seed, leaf	Decoction, raw	Gastric pains	3	0.012	1	0.004	
*Quercus ilex* L.	Brak	Infusion	Gastric pains	1	0.004	1	0.004	
*Quercus suber* L.	Fruit, bark, leaf	Decoction, cataplasm, cooked	Hypoglycemic, spasmolytic, skin wounds	3	0.012	3	0.012	
*Quercus rotundifolia* Lam.	Leaf	Infusion	Gastric pains	1	0.004	1	0.004	
Juglandaceae								0.020
*Juglans regia* L.	Brak, rhizome	Infusion, raw	Osteoarticular diseases, moth infections, intestinal pains, tooth care, dental hygiene	8	0.032	5	0.020	
Lamiaceae								0.026
*Rosmarinus officinalis* L.	Stem, flowers, leaf, whole plant	Infusion, decoction	Rheumatic pains, stomachic, gastric pains, anti-inflammatory, intestinal pains, hypotensive, spasmolytic, respiratory infections, cold, urinary infections, cutaneous infections	16	0.064	11	0.044	
*Thymus algeriensis* Boiss. & Reut.	Leaf, whole plant	Decoction, cataplasm	Skin wounds, respiratory infections, spasmolytic	4	0.016	3	0.012	
*Thymus serpyllum* L.	Flowers, leaf, whole plant	Infusion, decoction cataplasm, raw	Respiratory infections, spasmolytic, analgesic, stomachic, hypotensive, gastric pains, hypoglycemic, intestinal pains, cold, rheumatic pains, asthma	35	0.139	11	0.044	
*Thymus vulgaris*	Leaf	Decoction	Rheumatic pains, intestinal pains, respiratory infections	3	0.012	3	0.012	
*Ajuga iva* (L.) Schreb	Whole plant	Infusion	Rheumatic pains	1	0.004	1	0.004	
*Mentha* × *rotundifolia* (L.) Huds.	Leaf	Infusion, decoction	Asthma, respiratory infections	3	0.012	2	0.008	
*Mentha pulegium* L.	Stem, flowers, leaf	Infusion, decoction, cataplasm, cooked	Respiratory infections, stomachic, gastric pains, cold, laxative, intestinal pains, asthma, hypotensive	33	0.131	8	0.032	
*Lavandula x abrialis* L	Leaf, seed, whole plant	Infusion, decoction, cataplasm	Respiratory infections, rheumatic pains, spasmolytic, analgesic, skin wounds	8	0.032	5	0.020	
*Lavandula stoechas* L	Flowers, leaf, whole plant	Infusion, decoction	Stomachic, hypotensive, hypoglycemic, intestinal pains	7	0.028	4	0.016	
*Ocimum basilicum* L.	Whole plant	Cooked	Intestinal pains	1	0.004	1	0.004	
*Origanum compactum* Benth	Stem, flowers, leaf, whole plant	Infusion, decoction, raw	Intestinal pains, rheumatic pains, gastric pains, respiratory infections, hypoglycemic, urinary infections, spasmolytic, diabetes, stimulant, stomachic pains, diuretic	33	0.131	11	0.044	
*Origanum majorana* L.	Leaf	Infusion	Gastric pains	1	0.004	1	0.004	
*Mentha* × *piperita* L.	Flowers, whole plant, leaf	Infusion, decoction, cataplasm, cooked	Cold, digestive infections, diabetes, analgesic, skin wounds, cold, rheumatic pains, urinary infections	14	0.056	8	0.032	
*Marrubium vulgare* L.	Whole plant	Cataplasm	Respiratory infections	1	0.004	1	0.004	
*Melissa officinalis* L.	Stem, leaf	Infusion, decoction, cataplasm	Respiratory infections, rheumatic pains	3	0.012	2	0.008	
*Salvia officinalis* L.	Leaf	Decoction	Gastric pains, hypoglycemic, relaxant	3	0.012	3	0.012	
Lauraceae								0.004
*Cinnamomum verum* J.Presl	Brak	Infusion	Stimulant	1	0.004	1	0.004	
*Laurus nobilis* L.	Leaf	Infusion	Sedative	1	0.004	1	0.004	
Leguminosae								0.016
*Trigonella foenum-graecum* L.	Seed	Infusion, decoction	Hypotensive, gastric pains	3	0.012	2	0.008	
*Retama raetam* (Forssk.) Webb	Flowers, leaf	Decoction, cataplasm, raw	Dermatitis, skin ulcers, hypoglycemic, gastric pains, skin wounds, cutaneous infections	6	0.024	6	0.024	
Lythraceae								0.006
*Punica granatum* L.	Fruit, leaf	Infusion	Gastric pains	2	0.008	1	0.004	
*Lawsonia inermis* L.	Leaf	Infusion	Laxative, hair tonic	4	0.016	2	0.008	
Malvaceae								0.004
*Malva sylvestris* L.	Rhizome	Infusion	Haemorrhoids	1	0.004	1	0.004	
Moraceae								0.008
*Ficus carica* L.	Fruit	Raw	Laxative, hypotensive	3	0.012	2	0.008	
Myrtaceae								0.004
*Myrtus communis* L.	Whole plant	Cooked	Hair tonic	1	0.004	1	0.004	
Oleaceae								0.008
*Olea europaea* var. sylvestris (Mill.) Lehr	Fruit	Infusion, cataplasm	Hypoglycemic, cutaneous infections	2	0.008	2	0.008	
Parmeliaceae								0.004
*Evernia prunastri*	Whole plant	Cataplasm	Cutaneous infections	1	0.004	1	0.004	
Pinaceae								0.004
*Cedrus atlantica* (Endl.) Manetti ex Carrière	Leaf	Raw	Intestinal pains	1	0.004	1	0.004	
*Pinus pinaster* Aiton	Fruit	Infusion	Diabetes	1	0.004	1	0.004	
Plantaginaceae								0.004
*Plantago major* L	Leaf	Infusion	Skin wounds	1	0.004	1	0.004	
Poaceae								0.004
*Cynodon dactylon* (L.) Pers.	Whole plant	Infusion	Gastric pains	1	0.004	1	0.004	
*Pennisetum glaucum* (L.) R.Br.	Seed	Infusion	Osteoarticular diseases	1	0.004	1	0.004	
*Triticum aestivum* L.	Whole plant	Cooked	Skin ulcers	1	0.004	1	0.004	
Polygonaceae								0.004
*Emex spinosa* (L.) Campd	Rhizome	Decoction	Skin ulcers	1	0.004	1	0.004	
Ranunculaceae								0.008
*Nigella sativa* L.	Seed	Decoction	Hypotensive, urinair infections	2	0.008	2	0.008	
Rhamnaceae								0.012
*Ziziphus lotus* (L.) Lam	Leaf	Decoction	Rheumatic pains	1	0.004	1	0.004	
Rosaceae								
*Crataegus monogyna* Jacq.	Fruit, leaf	Decoction	Gastric pains	2	0.008	1	0.004	
*Rubus fruticosus* G.N.Jones	Fruit, leaf, brak	Decoction	Hair tonic, gastric pains, skin wounds, hypoglycemia, respiratory infections, skin ulcers	10	0.040	6	0.024	
*Prunus dulcis* (Mill.) D.A.Webb	Fruit	Cataplasm	Mixture for break bones	1	0.004	1	0.004	
*Prunus amygdaloides* Schltr.	Leaf	Infusion, raw	Respiratory infections, gastric pains, hair tonic	3	0.012	3	0.012	
Rutaceae								0.004
*Citrus limon* (L.) Osbeck	Seed	Cooked	Skin ulcer	1	0.004	1	0.004	
*Ruta chalepensis* L.	Leaf	Infusion	Gastric pains	1	0.004	1	0.004	
Salicaceae								0.004
*Populus alba* L.	Leaf	Decoction	Skin ulcer	1	0.004	1	0.004	
*Salix alba* L.	Leaf	Infusion	Gastric pains	1	0.004	1	0.004	
Solanaceae								0.004
*Atropa belladona* L.	Leaf	Infusion	Gastric pains	1	0.004	1	0.004	
*Hyoscyamus albus* L.	Seed	Cooked	Sedative	1	0.004	1	0.004	
*Solanum melongena* L.	Fruit	Cooked	Hypotensive	1	0.004	1	0.004	
Styracaceae								0.004
*Styrax benzoin* Dryand.	Stem	Cataplasm	Rheumatic pains	1	0.004	1	0.004	
Thymelaeaceae								0.024
*Daphne gnidium* L.	Flowers, bark, leaf, whole plant	Infusion, decoction, cataplasm, raw	Hair tonic, sedative, stomachic, gastric pains, respiratory infections, skin wounds	10	0.040	6	0.024	
Urticaceae								0.020
*Urtica dioica* L.	Leaf, whole plant	Decoction, cataplasm	Laxative, hair tonic, skin wounds, rheumatic pains, kidney stones	5	0.020	5	0.020	
Verbenaceae								0.024
*Aloysia citriodora* Palau	Leaf	Infusion, decoction	Hypoglycemic, gastric pains, spasmolytic, stimulant, diabetes, respiratory infections	7	0.028	6	0.024	
Xanthorrhoeaceae								0.004
*Asphodelus ramosus* L.	Bulb	Raw	Eczema	1	0.004	1	0.004	
Zygophyllaceae								0.004
*Etraena gaetula* (Emb. & Maire) Beier & Thulin	Flowers	Cataplasm	Cutaneous infections	1	0.004	1	0.004	

**Table 3:** Informants' consensus on the use of medicinal plants.

Category of ailments treated	Number of taxa (*N*_*t*_)	Use report (*N*_*ur*_)	ICF
Digestive diseases	43	111	0.618
Respiratory diseases	18	38	0.541
Skeletomuscular diseases	20	38	0.486
Dermatological diseases	31	53	0.423
Endocrine, metabolic, and nutritional diseases	21	31	0.333
Urological diseases	12	17	0.313
Cardiovascular diseases	15	19	0.222
Nervous system diseases	14	16	0.133

**Table 4:** Fidelity level of species used by the local population of Ketama.

Disease category	Species and fidelity level
Digestive diseases	*Anacyclus pyrethrum* (L.) Lag. (100), *Arbutus unedo* L. (100), *Atropa belladona* L. (100), *Cedrus atlantica* (Endl.) Manetti ex Carrière (100), *Cistus salviifolius* L. (100), *Crataegus monogyna* Jacq. (100), *Ocimum basilicum* L. (100), *Origanum majorana* L. (100), *Quercus ilex* L. (100), *Quercus rotundifolia* Lam. (100%), *Ruta chalepensis* L. (100), *Salix alba* L. (100), *Scolymus hispanicus* L. (100%), *Trigonella foenum-graecum* L. (83.33%), *Ficus carica* L. (66.67%), *Petroselinum crispum* (Mill.) Fuss (66.67%), *Origanum compactum* Benth (51.43%), *Artemisia arborescens* (Vaill.) L. (50%), *Cynodon dactylon* (L.) Pers. (50%), *Cistus albidus* L. (46.15%), *Rosmarinus officinalis* L. (43.75%), *Aloysia citriodora* Palau (42.86%), *Lavandula stoechas* L (42.86%), *Thymus serpyllum* L. (39.47%), *Mentha pulegium* L. (39.39%), *Cistus ladanifer* L. (39.33%), *Cistus laurifolius* L. (39.33%), *Opuntia ficus-indica* (L.) Mill. (39.33%), *Prunus amygdaloides* Schltr. (39.33%), *Quercus faginea* Lam. (39.33%), *Quercus suber* L. (39.33%), *Daphne gnidium* L. (30%), *Rubus fruticosus* G.N.Jones. (30%), *Artemisia herba-alba* Asso. (28.57%), *Juglans regia* L. (25%), *Lawsonia inermis* L. (25%), *Salvia officinalis* L. (25%), *Thymus algeriensis* Boiss. & Reut. (25%), *Nerium oleander* L. (16.67%), *Retama raetam* (Forssk.) Webb. (16.67%), *Urtica dioica* L. (16.67%), *Lavandula x abrialis* L. (14.29%), *Mentha rotundifolia* (L.) Huds. (9.09%), *Dittrichia viscosa* (L.) Greuter. (7.14%)

Respiratory diseases	*Marrubium vulgare* L. (100%), *Mentha* × *rotundifolia* (L.) Huds. (100%) *Thymus vulgaris* L. (100%), *Melissa officinalis* L. (66.66%), *Mentha pulegium* L. (42.42%), *Prunus amygdaloides* Schltr. (33.33%), *Cistus albidus* L. (30.76%), *Thymus algeriensis* Boiss. & Reut. (25%), *Daphne gnidium* L. (20%), *Origanum compactum* Benth. (17.14%), *Dittrichia viscosa* (L.) Greuter. (14.28%), *Thymus serpyllum* L. (14.28%), *Aloysia citriodora* Palau (13.15%), *Rosmarinus officinalis* L. (12.5%), *Mentha rotundifolia* (L.) Huds. (9.09%), *Cannabis sativa* L. (8.3%)

Skeletomuscular diseases	*Ajuga iva* (L.) Schreb (100%), *Cyanus triumfettii* (All.) (100%), *Foeniculum vulgare* Mill. (100%), *Pennisetum glaucum* (L.) R.Br. (100%), *Punica granatum* L. (100%), *Styrax benzoin* Dryand. (100%), *Ziziphus lotus* (L.) Lam (100%), *Lavandula x abrialis* L. (57.14%), *Mentha rotundifolia* (L.) Huds. (36.36%), *Melissa officinalis* L. (33.33%), *Juglans regia* L. (25%), *Rosmarinus officinalis* L. (18.75%), *Urtica dioica* L. (16.66%), *Nerium oleander* L. (16.66%), *Artemisia herba-alba* Asso. (14.28%), *Origanum compactum* Benth. (11.42%), *Thymus serpyllum* L. (10.52%), *Dittrichia viscosa* (L.) Greuter. (7.14%)

Dermatological diseases	*Asphodelus ramosus* L. (100%), *Citrus limon* (L.) Osbeck (100%), *Emex spinosa* (L.) Campd (100%), *Etraena gaetula* (Emb. & Maire) Beier & Thulin (100%), *Evernia prunastri* (100%), *Myrtus communis* L. (100%), *Plantago major* L. (100%), *Tetraclinis articulata* (Vahl) Mast. (100%), *Triticum aestivum* L. (100%), *Lawsonia inermis* L. (75%), *Aristolochia longa* L. (66.66%), *Cannabis sativa* L. (66.66%). *Retama raetam* (Forssk.) Webb (66.66%), *Dittrichia viscosa* (L.) Greuter (64.28%), *Allium sativum* L. (50%), *Nerium oleander* L. (50%), *Olea europaea* var. sylvestris (Mill.) Lehr (50%), *Artemisia herba-alba* Asso. (42.85%), *Daphne gnidium* L. (40%), *Opuntia ficus-indica* (L.) Mill. (33.33%), *Prunus amygdaloides* Schltr. (33.33%), *Quercus faginea* Lam. (33.33%), *Quercus suber* L. (33.33%), *Urtica dioica* L. (33.33%), *Lavandula x abrialis* L. (30%), *Thymus algeriensis* Boiss. & Reut. (28.57%), *Rubus fruticosus* G.N.Jones (25%), *Juglans regia* L. (12.5%), *Rosmarinus officinalis* L. (12.5%), *Mentha rotundifolia* (L.) Huds. (9.09%), *Cistus albidus* L. (7.69%), *Thymus serpyllum* L. (2.63%)

Endocrine, metabolic, and nutritional diseases	*Cinnamomum verum* J.Presl (100%), *Malva sylvestris L*. (100%), *Pinus pinaster* Aiton (100%), *Olea europaea* var. sylvestris (Mill.) Lehr (50%), *Salvia officinalis* L. (50%), *Rubus fruticosus* G.N.Jones(40%), *Aristolochia longa* L. (33.33%), *Cistus laurifolius* L. (33.33%), *Quercus faginea* Lam. (33.33%), *Quercus suber* L. (33.33%), *Aloysia citriodora* Palau (28.57%), *Thymus algeriensis* Boiss. & Reut. (25%), *Juglans regia* L. (25%), *Retama raetam* (Forssk.) Webb. (16.66%), *Lavandula stoechas* L. (14.28%), *Thymus serpyllum* L. (13.15%), *Mentha rotundifolia* (L.) Huds. (9.09%), *Origanum compactum* Benth. (8.57%), *Cannabis sativa* L. (8.33%), *Cistus albidus* L. (7.69%), *Mentha pulegium* L. (3.03%)

Urological diseases	*Herniaria hirsuta* L. (100%), *Populus alba* L. (100%), *Nigella sativa* L. (50%), *Opuntia ficus-indica* (L.) Mill. (33.33%), *Urtica dioica* L. (33.33%), *Petroselinum crispum* (Mill.) Fuss (33.33%), *Lavandula stoechas* L. (28.57%), *Juglans regia* L. (12.5%), *Mentha pulegium* L. (9.09%), *Mentha rotundifolia* (L.) Huds (9.09%), *Origanum compactum* Benth (8.57%), *Rosmarinus officinalis* L. (6.25%), *Thymus serpyllum* L. (2.63%)

Cardiovascular diseases	*Allium cepa* L. (100%), *Solanum melongena* L. (100%), *Cistus ladanifer* L (66.66%), *Nigella sativa* L. (50%), *Ficus carica* L. (33.33%), *Cistus laurifolius* L. (33.33%), *Trigonella foenum-graecum* L. (16.66%), *Artemisia herba-alba* Asso (14.28%), *Lavandula stoechas* L. (14.28%), *Thymus serpyllum* L. (10.52%), *Cannabis sativa* L. (8.33%), *Cistus albidus* L. (7.69%), *Dittrichia viscosa* (L.) Greuter (7.14%), *Rosmarinus officinalis* L. (6.25%), *Mentha pulegium* L. (3.03%)

Nervous system diseases	*Dysphania ambrosioides* (L.) Mosyakin & Clemants (100%), *Hyoscyamus albus* L. (100%), *Laurus nobilis* L. (100%), *Urginea maritima* (L.) Baker (100%), *Artemisia arborescens* (Vaill.) L. (50%), *Cynodon dactylon* (L.) Pers. (50%), *Allium sativum* L. (50%), *Mentha rotundifolia* (L.) Huds. (18.18%), *Nerium oleander* L. (16.66%), *Aloysia citriodora* Palau (14.28%), *Daphne gnidium* L. (10%), *Cannabis sativa* L. (8.33%), *Mentha pulegium* L. (3.03%), *Origanum compactum* Benth (2.8%)

**Table 5:** Jaccard similarity index for Ketama and other studies in neighbouring areas.

Area	Study year	Number of recorded plant species	Total species common in both areas	Species enlisted only in the study area	Species enlisted only in the aligned area	Plants with similar uses	Plants with dissimilar uses	Jaccard index	References
(1) Talassemtane National Park Morocco	2019	13	1	80	12	7.69	92.30	1.09	[48]
(2) Taounate Morocco	2003	102	32	49	70	31.37	68.62	36.78	[31]
(3) Rif Morocco	2018	30	4	77	26	13.33	86.66	4.04	[7]
(4) Rif Morocco	2016	280	43	38	237	15.35	84.64	18.53	[8]
(5) Middle Atlas Morocco	2019	35	14	67	21	40	60	18.91	[26]
(6) Taza Morocco	2020	46	15	66	31	32.60	67.39	18.29	[49]
(7) Oriental Morocco	2014	148	34	47	114	22.97	77.02	26.77	[40]
(8) Rif Morocco	2016	33	5	76	28	15.15	84.84	5.05	[24]
(9) Ksar Lakbir Morocco	1998	186	36	45	150	19.35	80.64	22.64	[6]
(10) Taza Morocco	2021	40	13	68	27	32.5	67.5	15.85	[34]
Average		91.3	19.7	61.3	71.6	23.03	76.96	16.79	

## Data Availability

No data were used in this study.
